# Association of parity with the timing and type of menopause: a longitudinal cohort study

**DOI:** 10.1093/aje/kwae320

**Published:** 2024-08-28

**Authors:** Natalie V Scime, Beili Huang, Hilary K Brown, Erin A Brennand

**Affiliations:** Department of Health and Society, University of Toronto Scarborough, Toronto, ON, Canada; Department of Obstetrics and Gynecology, University of Calgary, Calgary, AB, Canada; Department of Health and Society, University of Toronto Scarborough, Toronto, ON, Canada; Dalla Lana School of Public Health, University of Toronto, Toronto, ON, Canada; Department of Obstetrics and Gynecology, University of Calgary, Calgary, AB, Canada; Department of Community Health Sciences, University of Calgary, Calgary, AB, Canada

**Keywords:** menopause, oophorectomy, hysterectomy, parity, longitudinal study, childbirth, Alberta’s tomorrow project

## Abstract

We investigated the time-varying association between parity and timing of natural menopause, surgical menopause, and premenopausal hysterectomy among 23 728 women aged 40-65 years at enrollment in the Alberta’s Tomorrow Project cohort study (2000-2022), using flexible parametric survival analysis. Overall, natural menopause was most common by study end (57.2%), followed by premenopausal hysterectomy (11.4%) and surgical menopause (5.3%). Risks of natural menopause before age 50 years were elevated for 0 births (adjusted hazard ratio [aHR] at age 45, 1.33; 95% CI, 1.18-1.49) and 1 birth (aHR age 45, 1.21; 95% CI, 1.07-1.38), but similar for ≥3 births (aHR age 45, 0.95; 95% CI, 0.85-1.06) compared to 2 births (reference). Elevated risks of surgical menopause before age 45 years for 0 births (aHR age 40, 1.37; 95% CI, 1.09-1.69) and 1 birth (aHR age 40, 1.11; 95% CI, 0.85-1.45) attenuated when excluding women with past infertility or recurrent pregnancy loss, and reduced risks were observed over time for ≥3 births (aHR age 50, 0.84; 95% CI, 0.75-0.94). Risks of premenopausal hysterectomy were lower before age 50 years for 0 births (aHR age 45, 0.82; 95% CI, 0.76-0.88) but elevated after age 40 years for ≥3 births (aHR age 50, 1.25; 95% CI, 1.08-1.45). These complex associations necessitate additional research on the sociobiological impacts of childbearing on gynecologic health.

## Introduction

Menopause signals the end of female reproductive and ovarian function and is an important marker of health and chronic disease risk in women. Natural menopause is defined by a final menstrual period (FMP) after 12 months of amenorrhea without obvious medical cause and occurs for most North American women between 45 and 58 years (mean age of 52 years).^1^ Departures from this biological norm, however, are common. Early menopause before age 45 affects an estimated 11%-14% of women and is associated with higher risks of cardiometabolic diseases, depression, osteoporosis, and premature death.^2^^-^^6^ Surgical menopause (removal of ovaries by oophorectomy) and premenopausal hysterectomy collectively affect approximately 20% of women and are associated with elevated risks of cardiovascular disease and dementia.^7^^-^^9^ Evidence on the determinants of menopause characteristics is thus of high public health and clinical interest to support healthy aging.

Parity appears to have an important influence on menopause. A meta-analysis of 9 cohort studies published up to 2017 found delayed timing of natural menopause for women with 1 or more live births compared to 0 births, with a pooled hazard ratio of 0.79 (95% CI, 0.71-0.87).^10^ Given the fixed nature of a female’s ovarian reserve, the prevailing “follicle sparing” hypothesis posits that childbearing-related changes in endogenous hormones, frequency of ovulation, and other regulators of ovarian activity may slow the rate of follicle decline and delay the onset of natural menopause.^11^^-^^13^

However, important nuances in the association of parity and menopause have not been fully elucidated. First, population-level data are not always consistent with the follicle sparing hypothesis when parity is analyzed as an ordered variable; that is, increasingly delayed timing of menopause is not observed beyond 2 or 3 births.^14^^,^^15^ Second, the predominant use of the Cox proportional hazards model, which assumes a constant hazard ratio over time, may be obfuscating how the effect of parity on menopause differs as women age. For example, a pooled analysis of 9 women’s health studies showed that the association between 0 or 1 birth (vs 2 or more births) and earlier timing of natural menopause was evident only when age at FMP was younger than 50 years but not when FMP was at or older than 50 years.^16^ Third, the associations between parity and medical types of menopause have been incompletely studied, despite potential bidirectional pathways between number of births and gynecologic conditions typically treated with oophorectomy or hysterectomy. For example, uterine fibroids or endometriosis may impair fertility and result in lower order parity,^17^^,^^18^ while higher order parity may increase the risk of pelvic organ prolapse later in life.^19^ We sought to address these nuances by investigating the association between parity and the timing and type of menopause in midlife women using a flexible survival analysis approach.

## Methods

### Study sample

We conducted a secondary analysis of the Alberta’s Tomorrow Project (ATP), a province-wide prospective cohort study investigating the etiology and healthcare utilization related to cancer and chronic diseases.^20^ A total of 52 810 Albertans (*n* = 34 950 females) aged 35-69 years with no history of cancer and the ability to communicate in English were recruited in 2 phases: 2-stage telephone random digit dialing mapped to regional health authorities (2000-2009); and volunteer sampling through several communication and advocacy strategies targeting pan-provincial (eg, partnerships with popular consumer loyalty programs) and local (eg, informational booths at hospitals) audiences (2009-2015). All participants provided written informed consent. We included female participants aged 40-65 years at baseline who provided data on parity and menopausal status, excluding those who were pregnant at baseline, reported an extreme age at menopause (≤35 or >65 years) or unspecified menopause type, or were missing covariate data.

The ATP Study was approved by the Health Research Ethics Board of Alberta at Alberta Innovates (HREBA.CC-17-0461 and HREBA.CC-17-0494). These secondary analyses of ATP data were approved by the Conjoint Health Research Ethics Board at the University of Calgary (REB22-0742).

### Data collection

Comprehensive health and demographic data were collected through standardized questionnaires at baseline and approximately every 3-5 years thereafter (response rates of 70-80%). For these analyses, we used self-reported data from baseline and all follow-up questionnaires completed by August 2022, ranging from 1 to 5 study contacts per participant depending on the year they enrolled.

### Measures

Parity, defined as number of births ≥20 weeks’ gestation, was measured at baseline. Women with 2 births, comprising the largest parity group in the sample, were used as the reference group and compared to women with 0, 1, and ≥3 births.

Menopause characteristics were measured at baseline and each follow-up through self-report of the experience and timing of an FMP, hysterectomy, or oophorectomy. Menopause type was defined as premenopause; natural menopause with an FMP for no medical cause or intervention; surgical menopause induced through bilateral oophorectomy; premenopausal hysterectomy with ovarian preservation, whereby loss of menstruation with intact ovaries renders the clinical timing of menopause inconclusive. Menopause timing was defined as age at FMP for natural menopause and age at time of surgery for surgical menopause and premenopausal hysterectomy.

Covariates were selected based on prior studies^14^^-^^16^ and measured at baseline through self-report. Demographic characteristics were participant year of birth and educational attainment (high school or less, college degree, university degree, or postgraduate degree). Health-related behaviors were smoking status (never, past, or current) and lifetime duration of hormonal contraceptive use (years, inclusive of zero for never users). Reproductive history included infertility (ever trying to become pregnant for more than 1 year without conception or use of fertility treatments) and number of pregnancy losses <20 weeks’ gestation (0, 1, or ≥2). Physical health factors were body mass index (BMI) and physician-diagnosed diabetes, cardiovascular disease (including hypertension), and autoimmune disease (eg, rheumatoid arthritis, inflammatory bowel disease, multiple sclerosis). Menopausal hormone therapy (MHT) was measured at baseline and most follow-up contacts and defined as lifetime use (never, ever), as well as timing of initiation (never, premenopausal, postmenopausal, unknown).

### Analysis

We analyzed the association between parity and timing of menopause using flexible parametric survival analysis. We modeled menopause type–specific hazards to account for competing risks given that menopause can only occur due to a single cause.^21^ With age as the time scale, person-time at risk was counted in years from age 35 to age at menopause or censoring. Censoring events were the earliest of end of study follow-up, attrition, the occurrence of a competing menopause type, or reaching age 65 years (age 60 was used for modeling of surgical menopause and premenopausal hysterectomy owing to small event counts by parity group thereafter). We allowed this association to vary over time using restricted cubic splines with 4 degrees of freedom (3 internal knots) for the baseline hazard and 1 degree of freedom (no internal knots, a linear function of log time on the log cumulative hazards scale) for the effect of parity. First, we estimated crude cumulative incidence functions with simulation-based 95% confidence intervals (CIs), representing the probability of experiencing a given menopause type before a given time and before the occurrence of a competing menopause type among each parity group.^21^^,^^22^ Next, we estimated hazard ratios (HRs) and 95% CIs for earlier menopause, unadjusted and adjusted for birth year, education, smoking, and duration of hormonal contraceptive use. An HR greater than 1 indicated earlier menopause in the comparator group (0, 1, or ≥3 births) than the reference group (2 births) among those who were still premenopausal up to a given time point. We plotted age-specific adjusted HR curves and reported numerical adjusted HRs in 5-year increments, regardless of HR curve trends, to correspond to ages that are clinically meaningful, facilitate comparison across models, and limit selective reporting of “statistically significant” results.

Among women who experienced menopause between 35 and 65 years, we analyzed the association between parity and type of menopause using multinomial logistic regression, with natural menopause as the reference outcome group. We estimated odds ratios (ORs) and 95% CIs, unadjusted and adjusted for birth year, education, smoking, and duration of hormonal contraceptive use.

We conducted 5 sensitivity analyses. First, we explored whether the associations differed when restricted to women without history of infertility or recurrent (≥2) pregnancy loss. Second, we accounted for the potential influence of MHT use prior to menopause, which can impact vaginal bleeding patterns, by adding premenopausal MHT (based on self-reported age at initiation) use as a censoring event for the survival models and excluding women reporting premenopausal MHT use from the multinomial models. Third, we further adjusted for baseline BMI and chronic medical conditions, which could have confounded the associations of interest but may not have temporally preceded both exposure and outcome depending on each woman’s health trajectory and age at enrollment. Fourth, we explored potential reverse causation, wherein a shorter reproductive window given early onset of menopause could have systematically reduced parity, by restricting to women who experienced menopause at >40 years, at which point the majority of parous women have completed childbearing.^23^ Fifth, for survival models only, we accounted for potential informative censoring using stabilized inverse probability of censoring weights.^24^^,^^25^ Weights for each age year analyzed were calculated using pooled logistic regression as the probability of remaining uncensored conditional on parity divided by the probability of remaining uncensored conditional on parity, birth year, baseline age, education, smoking, infertility, pregnancy losses, duration of hormonal contraceptive use, baseline BMI, and chronic medical conditions; weights were then multiplied cumulatively across time for each participant.^26^

Data were cleaned in Stata MP version 17 and analyzed and visualized in R version 4.2.2.^27^^,^^28^

## Results

Of the 23 728 females analyzed (Figure S1), 16.6% were nulliparous, 11.7% reported 1 birth, 40.9% reported 2 births, and 30.8% reported ≥3 births. The proportions of women reporting a college degree or high school diploma or less, former or current smoking, and ever using hormonal contraceptives were larger in higher order parity groups (Table 1). Pregnancy loss and infertility were most frequent in women with 1 birth. Baseline BMI, chronic medical conditions, and MHT use were fairly similar across parity groups.

**Table 1:** Baseline characteristics of Alberta’s Tomorrow Project female participants by parity.

**Characteristic**	**0 Births** ***n* = 3942**		**1 Birth** ***n* = 2787**		**2 Births** ***n* = 9695**		**≥3 Births** ***n* = 7304**	
	**n**	**%**	**n**	**%**	**n**	**%**	**n**	**%**
Birth year								
1930s	26	0.7	22	0.8	83	0.9	171	2.3
1940s	516	13.1	443	15.9	1705	17.6	1725	23.6
1950s	1654	42.0	1208	43.3	4523	46.7	3415	46.8
1960s	1485	37.7	962	34.5	2987	30.8	1810	24.8
1970s	261	6.6	152	5.5	397	4.1	183	2.5
Age at baseline, mean (SD)	50.8	(6.7)	51.1	(6.9)	51.7	(6.8)	52.8	(6.9)
Education								
High school or less	1012	25.7	967	34.7	3448	35.6	3194	43.7
College degree	1087	27.6	806	28.9	2941	30.3	2168	29.7
University degree	1171	29.7	705	25.3	2459	25.4	1543	21.1
Post-graduate degree	672	17.0	309	11.1	847	8.7	399	5.5
Pregnancy loss								
0	2980	75.6	1663	59.7	6262	64.6	4432	60.7
1	648	16.4	695	24.9	2368	24.4	1880	25.7
≥2	314	8.0	429	15.4	1065	11.0	992	13.6
Infertility								
No	3337	85.3	2185	78.5	8527	88.0	6553	89.8
Yes	576	14.7	600	21.5	1160	12.0	746	10.2
Smoking status								
Never	2187	55.5	1274	45.7	4779	49.3	3747	51.3
Former	1305	33.1	1090	39.1	3792	39.1	2655	36.3
Current	450	11.4	423	15.2	1124	11.6	902	12.3
Hormonal contraceptive use								
Never used	665	16.9	234	8.4	680	7.0	783	10.7
Ever used	3277	83.1	2553	91.6	9015	93.0	6521	89.3
Years of use, mean (SD)	9.7	(7.6)	8.2	(6.3)	7.4	(5.4)	5.9	(4.3)
Body mass index, mean (SD)	27.5	(6.9)	27.4	(6.3)	26.8	(5.6)	27.4	(5.8)
Chronic medical conditions								
Diabetes	166	4.2	160	5.8	487	5.0	375	5.2
Cardiovascular disease	854	21.7	660	23.7	2148	22.2	1845	25.3
Autoimmune disease	393	10.0	241	8.7	788	8.2	601	8.3
Menopausal hormone therapy								
Never used	2653	67.3	1821	65.4	6215	64.1	4590	62.9
Ever used	1288	32.7	965	34.6	3476	35.9	2711	37.1
Premenopausal initiation	538	42.2	367	38.3	1360	39.3	1001	37.1
Postmenopausal initiation	579	45.4	458	47.9	1629	47.1	1256	46.5
Initiation timing unknown	158	12.4	132	13.8	469	13.6	442	16.4

Abbreviation: SD: standard deviation.

**Figure 1:**
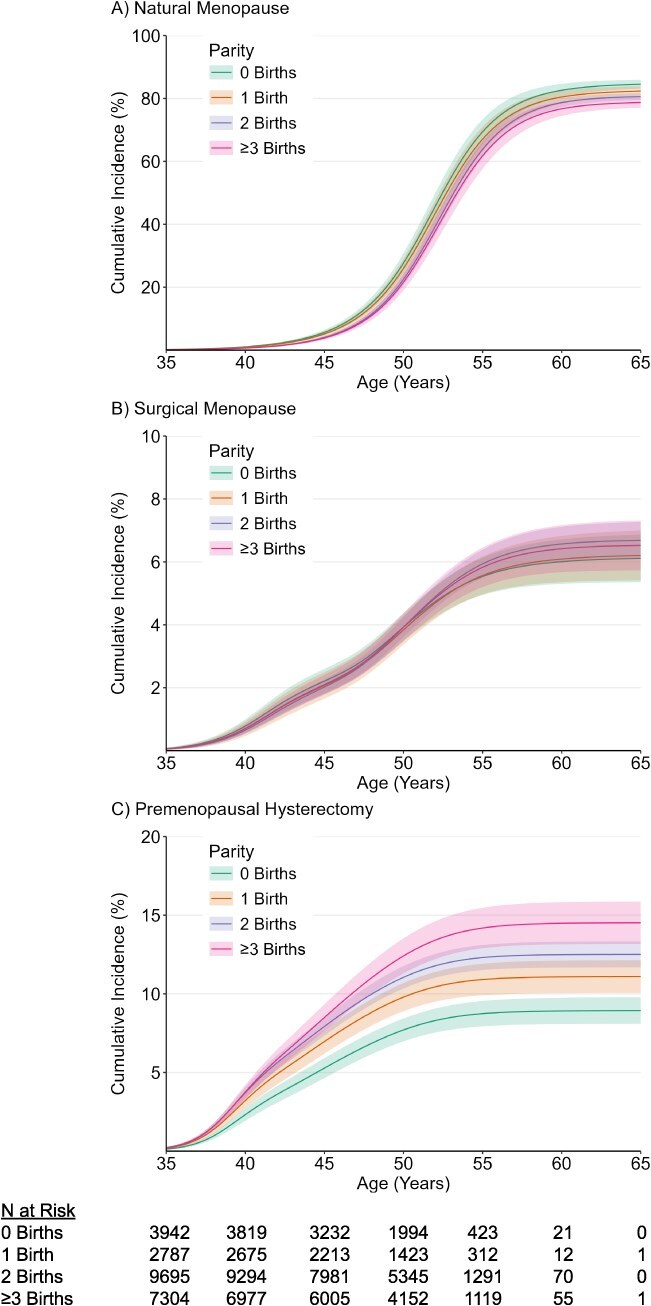
Cumulative incidence of natural menopause, surgical menopause, and premenopausal hysterectomy by parity. Y-axes of panel B and C were resized for better visualization. The number of participants at risk at each timepoint excludes participants who experienced menopause and participants who were censored premenopausally.

By the end of the study period, 73.9% of women experienced menopause; natural menopause was most frequent (57.2%), followed by premenopausal hysterectomy (11.4%) and surgical menopause (5.3%). For natural menopause, a negative gradient with higher cumulative incidence curves among lower order parity groups was observed (Figure 1). Conversely, for surgical menopause and premenopausal hysterectomy, a positive gradient with higher cumulative incidence curve among higher order parity groups was observed; curves were more distinct with nonoverlapping 95% CI, for premenopausal hysterectomy (Figure 1). Crude median and interquartile range for age at each menopause type by parity group showed slight rightward shifts in the distribution of timing for natural and surgical menopause with higher order parity (Table S1).

**Figure 2:**
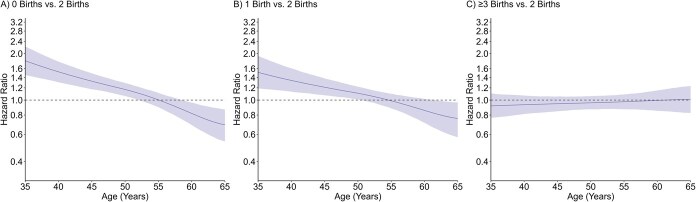
Adjusted association of parity and timing of natural menopause. Models controlled for birth year, education, smoking, and duration of hormonal contraceptive use.

**Figure 3:**
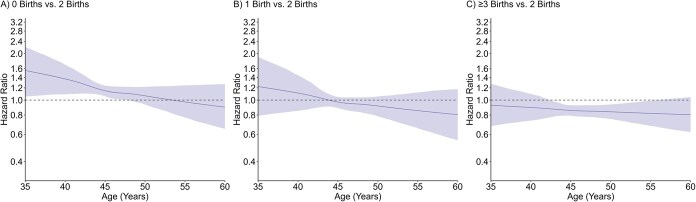
Adjusted association of parity and timing of surgical menopause. Models controlled for birth year, education, smoking, and duration of hormonal contraceptive use.

**Figure 4:**
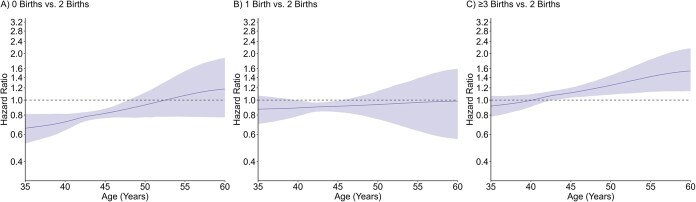
Adjusted association of parity and timing of premenopausal hysterectomy. Models controlled for birth year, education, smoking, and duration of hormonal contraceptive use.

Parity was associated with timing of all types of menopause in crude and adjusted models in a time-varying manner, with no clear evidence of a monotonic dose–response effect. For natural menopause (Figure 2, Table S2), adjusted HRs between approximately ages 35 and 50 years indicated higher risk of earlier natural menopause in women with 0 births (age 45, 1.33; 95% CI, 1.18-1.49) and 1 birth (age 45, 1.21; 95% CI, 1.07-1.38) compared to 2 births during this age interval, but there were no association thereafter. No evidence of an association was observed between higher order parity and timing of natural menopause. For surgical menopause (Figure 3, Table S2), adjusted HRs between approximately ages 35 and 45 years indicated higher risk of earlier surgical menopause in lower order parity groups (age 40 for 0 births, 1.37; 95% CI, 1.09-1.69; 1 birth, 1.10, 95% CI ; 95% CI, 0.85-1.45), and adjusted HRs across follow-up indicated lower risk of surgical menopause in women with ≥3 births (age 50, 0.84; 95% CI, 0.75-0.94); however, 95% CIs were generally wide and enclosed the null value at age extremes. For premenopausal hysterectomy (Figure 4, Table S2), adjusted HRs between approximately ages 35 and 50 years indicated lower risk of premenopausal hysterectomy in women with 0 births (age 45, 0.82; 95% CI, 0.76-0.88) compared to 2 births during this age interval, but there was no association thereafter. Conversely, adjusted HRs after approximately age 45 years indicated higher risk of early premenopausal hysterectomy in women with ≥3 births (age 50, 1.25; 95% CI, 1.08-1.45) compared to 2 births. Adjusted HRs at the older age extremes generally had wide 95% CI.

Among women who experienced menopause, parity was associated with odds of premenopausal hysterectomy but not surgical menopause in crude and adjusted models using natural menopause as the reference outcome group (Table 2). Compared to women with 2 births, women with 0 births had lower odds of premenopausal hysterectomy (adjusted OR, 0.75; 95% CI, 0.65-0.86), whereas women with ≥3 births had higher odds of premenopausal hysterectomy (adjusted OR, 1.11; 95% CI, 1.01-1.22).

**Table 2:** Association of parity and menopause type among women who experienced menopause.

	**No., % with the outcome**						**Odds ratio (95% confidence interval)**							
							**Surgical**				**Hysterectomy**			
	**Natural (ref)**		**Surgical**		**Hysterectomy**		**Crude**		**Adjusted**		**Crude**		**Adjusted**	
0 Births	2223	(81.4)	192	(7.0)	317	(11.6)	0.91	(0.76-1.08)	1.06	(0.89-1.27)	0.70	(0.61-0.80)	0.75	(0.65-0.86)
1 Birth	1545	(78.8)	136	(6.9)	280	(14.3)	0.93	(0.76-1.13)	0.94	(0.77-1.15)	0.89	(0.77-1.03)	0.87	(0.76-1.01)
2 Births (ref)	5530	(77.0)	526	(7.3)	1125	(15.7)	1		1		1		1	
≥3 Births	4291	(75.6)	402	(7.1)	984	(17.3)	0.99	(0.86-1.13)	0.87	(0.76-1.00)	1.13	(1.03-1.24)	1.11	(1.01-1.22)

Adjusted models controlled for birth year, education, smoking, and duration of hormonal contraceptive use.

Results from the survival models (Figures S2, S3, S4) and multinomial logistic models (Table S3) were robust to sensitivity analyses censoring or excluding women at initiation of premenopausal MHT, additionally adjusting for baseline BMI and chronic medical conditions, as well as inverse probability of censoring weighting. Of note, exclusion of women with a history of infertility or recurrent pregnancy loss attenuated point estimates for the association between 1 birth and risk of early natural menopause between ages 35 and 50 years (adjusted HR at age 45, 1.16; 95% CI, 1.01-1.34); and the associations between 0 (age 40, 0.99; 95% CI, 0.74-1.33) and 1 (age 40, 0.90; 95% CI, 0.64-1.26) birth and risk of early surgical menopause before age 45 years. Restricting to women who experienced menopause at >40 years of age substantially decreased precision for the associations between parity and surgical menopause and premenopausal hysterectomy, though the magnitude of point estimates were similar.

## Discussion

In this community-based cohort study, we detected a complex and age-dependent relationship between parity and the timing and type of menopause. We found an elevated risk of earlier natural menopause before age 50 years in women with 0 or 1 birth but similar timing of natural menopause in women with ≥3 births compared to those with 2 births. We observed slightly higher risk of earlier surgical menopause before age 45 years in lower order parity groups, which was no longer evident in sensitivity analyses excluding women with a history of infertility or recurrent pregnancy loss. We also reported distinct associations between parity extremes and premenopausal hysterectomy, with reduced risk of premenopausal hysterectomy before age 50 years in women with 0 births and elevated risk of premenopausal hysterectomy after age 40 years in women with ≥3 births compared to those with 2 births.

In alignment with other studies, our results do not support the follicle sparing hypothesis (ie, a positive monotonic association between parity and progressively delayed age at menopause) as the sole mechanism for parity effects on natural menopause. A population study of 310 147 women in Norway found an L-shaped adjusted association between number of childbirths (from 0 to 7) and age at natural menopause, with no further delays in age at menopause with more than 3 births.^15^ A prospective analysis of 108 887 women in the US Nurses Health Study II also found an L-shaped adjusted association between parity (from 0 to 4 or more) and risk of early natural menopause before age 45 years, with no further protective effect beyond 3 births.^14^ Our results based on Canadian women similarly found that the adjusted association between parity (from 0 to 3 or more) and risk of early natural menopause before age 50 years was not evident beyond 2 births. Alternative mechanisms should therefore focus on elucidating the distinct gradient of risk for early natural menopause between parity of 0 to parity of 2 or 3, but not thereafter.

One explanation could be that lower order parity and early natural menopause reflect underlying early ovarian failure, resulting in some degree of reverse causation. That is, women destined to have earlier menopause achieve fewer births during the reproductive years because reduced ovarian function results in longer and less successful attempts to conceive,^29^ particularly through reduced likelihood of success with assisted reproductive technology.^30^ However, our results were fairly robust to exclusion of women with a history of infertility or recurrent pregnancy loss, and history of infertility defined broadly does not appear to be related to markers of ovarian reserve^31^ or timing of natural menopause.^32^ This mechanism would also be less relevant when intended and achieved parity are equal, as is the case for an estimated 40%-60% of women with 0 or 1 child.^33^ A more plausible explanation could involve a complex trade-off of physiological and social changes related to childbearing that impact ovarian aging. Nuanced discussion of such trade-offs have been considered in broader life course research,^34^^,^^35^ for example, to explain why parity has a U-shaped relationship with cellular aging^36^ and a J-shaped relationship with mortality risk^37^ that reach a minimum between 2 to 4 births. We propose that physiologic advantages of childbearing on ovarian capacity (ie, follicle sparing) may be counteracted by deleterious psychosocial processes that are pronounced when reproduction exceeds the social norm of 2 to 3 children. For example, a handful of cross-sectional studies suggest that higher psychological stress and reduced economic resources, which occur more frequently among women with 4 or more children,^38^^-^^41^ may be associated with reduced ovarian reserve.^42^^-^^44^ Future research on natural menopause could assess this proposition using a more holistic view of parity as a biological *and* social phenomenon; such as exploring effect heterogeneity by socioeconomic strata, analyzing latent classes of childbearing patterns or “biographies,”^39^ or incorporating measures of allostatic load (the cumulative physiological toll of exposure to social stressors)^45^ into analyses.

To our knowledge, our analysis of parity and medical types of menopause is a novel addition to the menopause epidemiology literature. Our results for premenopausal hysterectomy are congruent with a British prospective cohort study citing the lowest rate of 3.13 hysterectomies per 1000 nulliparous women and the highest hysterectomy rates in women with 3 or more births (adjusted HR, 2.79; 95% CI, 1.80-4.34); however, concomitant oophorectomy and menopause timing were not considered.^46^ Increasing parity is associated with reduced risk of uterine fibroids and endometriosis,^17^^,^^18^ which are indicated in approximately 20% of bilateral oophorectomies and 60% of hysterectomies in early adulthood.^47^^-^^49^ This is consistent with our finding of decreased risk of surgical menopause with higher order parity but paradoxical to our observation of decreased risk of premenopausal hysterectomy before age 50 years in nulliparous women. Replication and further investigation of this latter finding are needed.

Increasing risk of premenopausal hysterectomy after age 40 years in women with ≥3 births could be explained by the disproportionate incidence of certain gynecologic conditions in these women. Pelvic organ prolapse (POP) is an anatomic condition related to stretch and injury of pelvic floor muscles and ligaments and thus increasing parity, specifically vaginal birth, are established causal factors.^19^^,^^50^ POP is indicated in up to half of the hysterectomies performed at age 50 years or older,^47^^,^^48^^,^^51^ as the mainstay approach to surgical correction involves removing the uterus even when it is not among the affected organs.^52^ Some evidence suggests that increasing parity, and specifically Cesarean birth, are risk factors for abnormal uterine bleeding,^53^^,^^54^ which is a symptom indicated (sometimes in conjunction with established diagnoses) in roughly 20%-35% of hysterectomies performed at age 50 years or older.^47^^,^^48^^,^^51^ Future research should assess whether the association between high parity and premenopausal hysterectomy is indeed mediated by incidence of POP, abnormal bleeding, or other gynecologic conditions and explore this in relation to mode of delivery.

The main strengths of this analysis are the large community-based sample and detailed information on the type and timing of menopause that is not available in routinely collected (ie, health claims) data sources; however, limitations should be considered. Menopause characteristics were measured through a mix of retrospective and prospective data collection, depending on when women enrolled in the ATP Study. Women’s recall of menopause characteristics generally has moderate to high accuracy up to 20 years later^55^^-^^58^ yet is subject to memory error, and thus random misclassification error is possible. Residual confounding is likely given that some covariates (eg, educational attainment) were measured at baseline in midlife yet may have changed over the life course and differed during the childbearing years. This mistiming of covariate measurement also led us to exclude available covariates related to health behaviors (eg, alcohol use, physical activity) from our analyses, despite their inclusion in some studies on age at menopause. The modest number of events for surgical menopause and premenopausal hysterectomy sometimes hampered precision in adjusted models, wherein point estimates represented a clinically important difference in the timing of menopause yet 95% CIs were wide and included the null. Finally, although the ATP Study is largely representative of the Alberta female population, the use of volunteer sampling, restriction to English-speaking individuals, and unintentional undersampling of certain characteristics such as race/ethnic diversity and postsecondary education caveats the generalizability of our results.^20^

This cohort study found a complex relationship between parity and menopause that changes over the continuum of aging. Excess risks of earlier natural menopause before age 50 years were largest in nulliparous women followed by women with 1 birth compared to the reference group of women with 2 births, and with no differences in timing of natural menopause observed in women with 3 or more births. Parity extremes were also distinctly related to surgical menopause and premenopausal hysterectomy. Women with 0 or 1 birth had elevated risk of surgical menopause that may have been driven by prior infertility or recurrent pregnancy loss, and nulliparous women had reduced risk of premenopausal hysterectomy before age 50 years. Women with 3 or more births had uniformly reduced risk of surgical menopause and increasing risk of premenopausal hysterectomy after age 40 years. This work highlights several possible avenues for future research to advance a fuller view of the sociobiological impacts of childbearing on women’s menopausal transition and midlife health.

## Supplementary Material

Web_Material_kwae320

## Data Availability

Requests to access the data used in this study can be directed to the Alberta’s Tomorrow Project team at ATP.Research@albertahealthservices.ca.
