# Supplementary material for: Association of parity with the timing and type of menopause: a longitudinal cohort study
Source: Am J Epidemiol. 2024 Aug 28;194(6):1726–34. doi: 10.1093/aje/kwae320 (PMC12133278; doi:10.1093/aje/kwae320)
Supplement: Web_Material_kwae320 [file web_material_kwae320.zip › Web_Material.docx]

**Web Material**

**Association of parity with the timing and type of menopause: A longitudinal cohort study**

Natalie V. Scime, Beili Huang, Hilary K. Brown, Erin A. Brennand

**Contents:**

Tables S1-S3

Figures S1-S4

**Table S1. Median age at natural menopause, surgical menopause, and premenopausal hysterectomy by parity**

| Parity | Natural Menopause | | Surgical Menopause | | Premenopausal Hysterectomy | |
| --- | --- | --- | --- | --- | --- | --- |
|  | No. | Median (IQR) | No. | Median (IQR) | No. | Median (IQR) |
| 0 Births | 2220 | 51.0 (48.0, 53.0) | 192 | 45.0 (41.0, 50.0) | 317 | 43.0 (40.0, 46.0) |
| 1 Birth | 1542 | 51.0 (48.0, 53.0) | 136 | 46.5 (41.8, 51.2) | 280 | 42.0 (40.0, 46.0) |
| 2 Births | 5522 | 51.0 (49.0, 54.0) | 526 | 47.0 (42.2, 51.0) | 1125 | 43.0 (39.0, 46.0) |
| ≥3 Births | 4283 | 51.0 (49.0, 54.0) | 402 | 47.0 (43.0, 50.0) | 984 | 43.0 (40.0, 47.0) |

IQR: interquartile range.

**Table S2. Association of parity and timing of natural menopause, surgical menopause, and premenopausal hysterectomy**

| Parity | Hazard Ratio (95% Confidence Interval) | | | | | | | | | | | | | | | | | |
| --- | --- | --- | --- | --- | --- | --- | --- | --- | --- | --- | --- | --- | --- | --- | --- | --- | --- | --- |
|  | Crude | | | | | | | | Adjusted | | | | | | | | | |
|  | 40 Years | | 45 Years | | 50 Years | | 55 Years | | 40 Years | | | 45 Years | | | 50 Years | | 55 Years | |
| Natural Menopause | | | | | | | | | | | | | | | | | | |
| 0 Births | 1.47 | (1.25-1.70) | 1.29 | (1.15-1.44) | 1.15 | (1.04-1.26) | 1.00 | (0.90-1.12) | 1.52 | | (1.30-1.77) | 1.33 | | (1.18-1.49) | 1.17 | (1.06-1.29) | 1.00 | (0.90-1.11) |
| 1 Birth | 1.33 | (1.12-1.59) | 1.21 | (1.08-1.37) | 1.11 | (1.01-1.23) | 1.00 | (0.89-1.13) | 1.34 | | (1.13-1.61) | 1.21 | | (1.07-1.38) | 1.11 | (1.01-1.22) | 0.99 | (0.88-1.10) |
| 2 Births (ref) | 1 | – | 1 | – | 1 | – | 1 | – | 1 | | – | 1 | | – | 1 | – | 1 | – |
| ≥3 Births | 0.96 | (0.84-1.11) | 0.96 | (0.87-1.09) | 0.97 | (0.89-1.07) | 0.98 | (0.89-1.08) | 0.93 | | (0.81-1.07) | 0.95 | | (0.85-1.06) | 0.96 | (0.87-1.06) | 0.98 | (0.87-1.09) |
| Surgical Menopause | | | | | | | | | | | | | | | | | | |
| 0 Births | 1.12 | (0.91-1.40) | 0.93 | (0.84-1.01) | 0.89 | (0.78-1.00) | 0.81 | (0.64-1.01) | | 1.37 | (1.09-1.69) | | 1.15 | (1.06-1.24) | 1.07 | (0.94-1.22) | 0.97 | (0.77-1.24) |
| 1 Birth | 1.08 | (0.84-1.40) | 0.93 | (0.85-1.03) | 0.90 | (0.78-1.04) | 0.84 | (0.63-1.10) | | 1.11 | (0.85-1.45) | | 0.97 | (0.89-1.05) | 0.92 | (0.79-1.06) | 0.85 | (0.66-1.12) |
| 2 Births (ref) | 1 | – | 1 | – | 1 | – | 1 | – | | 1 | – | | 1 | – | 1 | – | 1 | – |
| ≥3 Births | 1.06 | (0.87-1.29) | 0.98 | (0.90-1.06) | 0.96 | (0.86-1.05) | 0.92 | (0.76-1.10) | | 0.90 | (0.74-1.10) | | 0.86 | (0.79-0.93) | 0.84 | (0.75-0.94) | 0.82 | (0.68-0.99) |
| Premenopausal hysterectomy | | | | | | | | | | | | | | | | | | |
| 0 Births | 0.65 | (0.58-0.73) | 0.74 | (0.68-0.80) | 0.84 | (0.68-1.00) | 0.96 | (0.68-1.35) | | 0.72 | (0.64-0.81) | | 0.82 | (0.76-0.88) | 0.93 | (0.77-1.12) | 1.07 | (0.78-1.54) |
| 1 Birth | 0.88 | (0.79-0.98) | 0.90 | (0.83-0.98) | 0.93 | (0.76-1.12) | 0.96 | (0.67-1.36) | | 0.89 | (0.79-1.00) | | 0.91 | (0.84-0.99) | 0.94 | (0.75-1.14) | 0.96 | (0.63-1.39) |
| 2 Births (ref) | 1 | – | 1 | – | 1 | – | 1 | – | | 1 | – | | 1 | – | 1 | – | 1 | – |
| ≥3 Births | 1.06 | (0.96-1.17) | 1.19 | (1.10-1.27) | 1.32 | (1.17-1.50) | 1.50 | (1.20-1.89) | | 1.00 | (0.90-1.09) | | 1.11 | (1.04-1.20) | 1.25 | (1.08-1.45) | 1.42 | (1.13-1.83) |

Adjusted models controlled for birth year, education, smoking, and duration of hormonal contraceptive use.

**Table S3. Sensitivity analyses of the adjusted association between parity and menopause type among women who experienced menopause**

| Sensitivity analysis | Parity | Adjusted Odds Ratio (95% Confidence Interval)  Reference Outcome Group: Natural | | | |
| --- | --- | --- | --- | --- | --- |
|  |  | Surgical Menopause | | Premenopausal Hysterectomy | |
| 1. Restricted to women without a history of infertility or recurrent pregnancy loss | 0 Births | 0.91 | (0.74-1.13) | 0.74 | (0.63-0.87) |
|  | 1 Birth | 0.87 | (0.68-1.12) | 0.89 | (0.75-1.05) |
|  | 2 Births (ref) | 1 | – | 1 | – |
|  | ≥3 Births | 0.86 | (0.73-1.00) | 1.07 | (0.96-1.20) |
| 2. Censored at initiation of premenopausal hormone therapy | 0 Births | 1.12 | (0.93-1.35) | 0.77 | (0.67-0.89) |
|  | 1 Birth | 0.99 | (0.80-1.23) | 0.89 | (0.76-1.03) |
|  | 2 Births (ref) | 1 | – | 1 | – |
|  | ≥3 Births | 0.87 | (0.75-1.01) | 1.10 | (1.00-1.22) |
| 3. Additionally adjusted for body mass index and chronic medical conditions | 0 Births | 1.02 | (0.85-1.23) | 0.73 | (0.64-0.85) |
|  | 1 Birth | 0.88 | (0.72-1.08) | 0.86 | (0.74-1.00) |
|  | 2 Births (ref) | 1 | – | 1 | – |
|  | ≥3 Births | 0.85 | (0.74-0.98) | 1.10 | (1.00-1.21) |
| 4. Restricted to menopause >40 years | 0 Births | 1.00 | (0.82-1.21) | 0.80 | (0.68-0.94) |
|  | 1 Birth | 0.87 | (0.70-1.09) | 0.86 | (0.72-1.02) |
|  | 2 Births (ref) | 1 | – | 1 | – |
|  | ≥3 Births | 0.90 | (0.78-1.05) | 1.19 | (1.06-1.34) |

Models controlled for birth year, education, smoking, and duration of hormonal contraceptive use. Fertility challenges were infertility and recurrent (≥ 2) pregnancy loss. Chronic medical conditions were diabetes, cardiovascular disease, and autoimmune disease.

**Figure S1. Flow diagram of included female participants from Alberta’s Tomorrow Project**

| 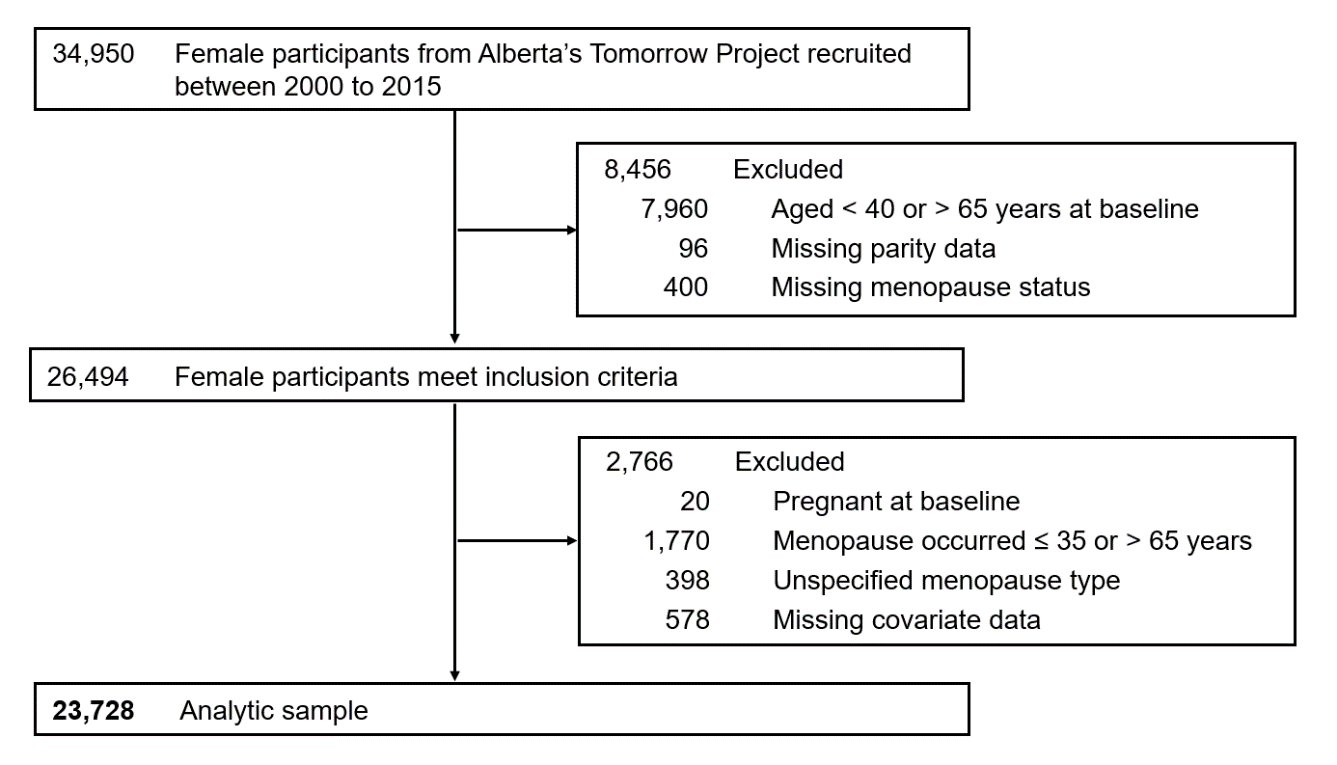 |
| --- |

**Figure S2. Sensitivity analyses of the adjusted association between parity and timing of natural menopause**

|  | A. 0 Births vs. 2 Births | B. 1 Birth vs. 2 Births | C. ≥3 Births vs. 2 Births |
| --- | --- | --- | --- |
| 1. Restricted to women without a history of infertility or recurrent pregnancy losses | 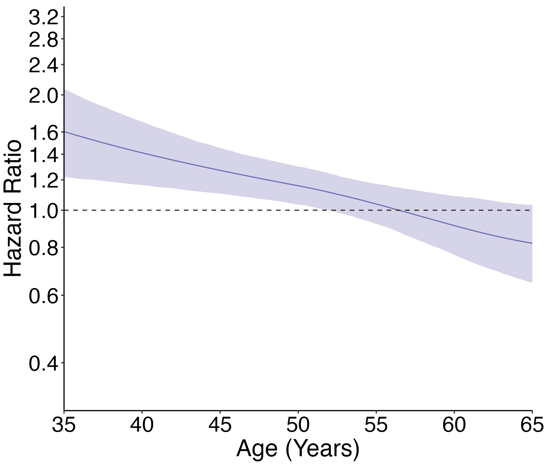 | 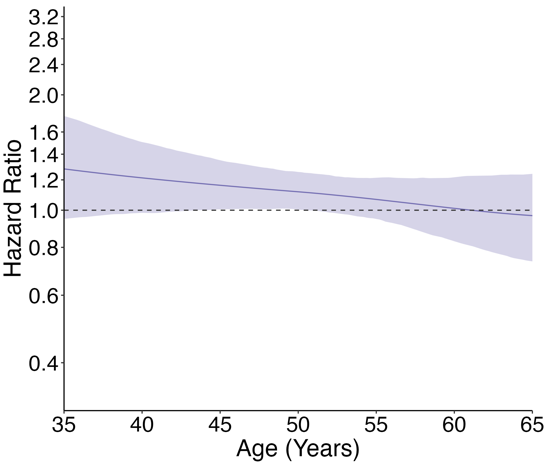 | 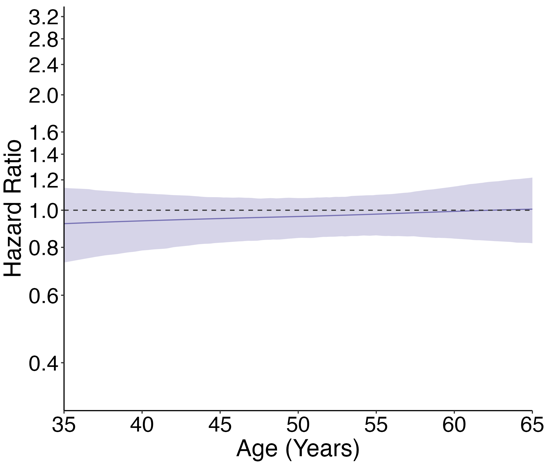 |
| 2. Censored at initiation of premenopausal hormone therapy | 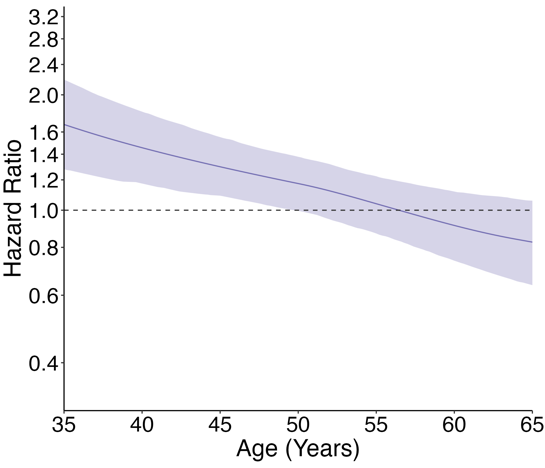 | 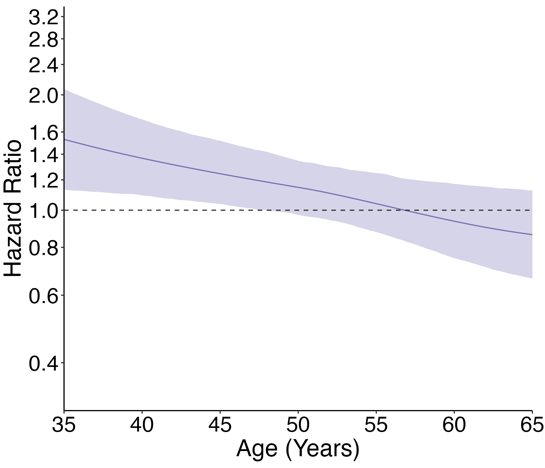 | 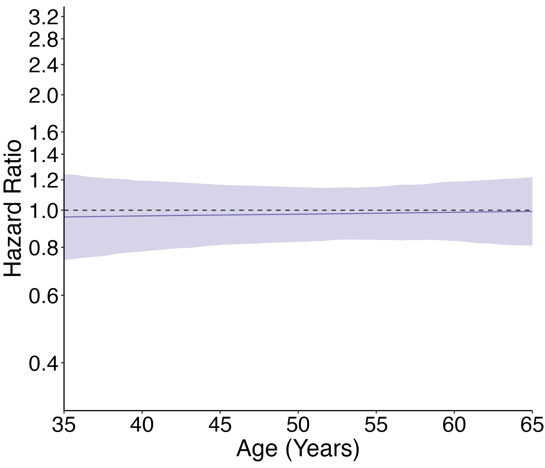 |
| 3. Additionally adjusted for body mass index and chronic medical conditions (diabetes, cardiovascular disease, autoimmune disease) | 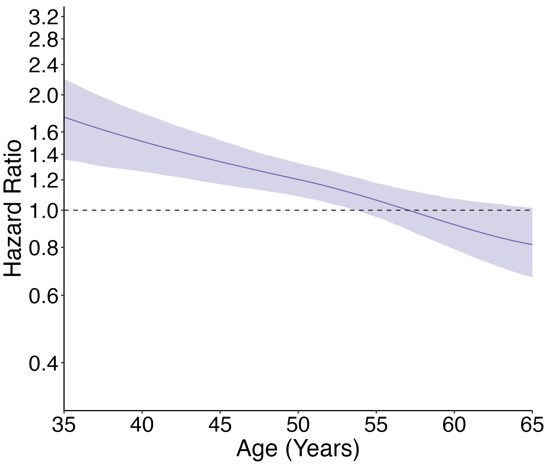 | 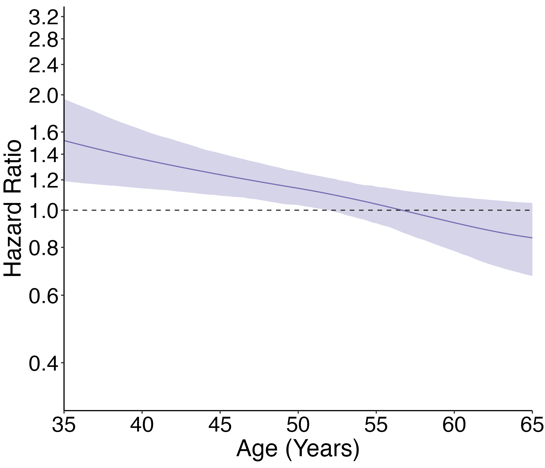 | 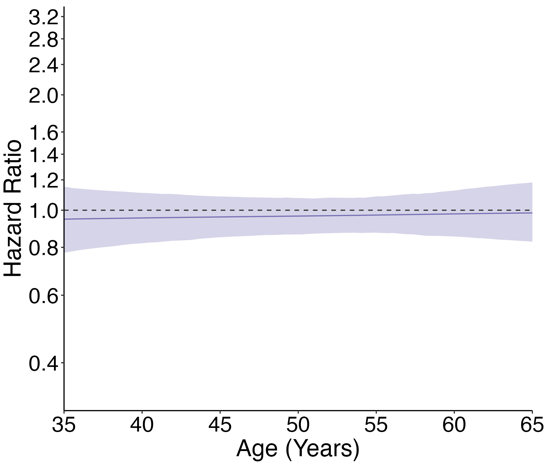 |
| 4. Restricted to menopause >40 years | 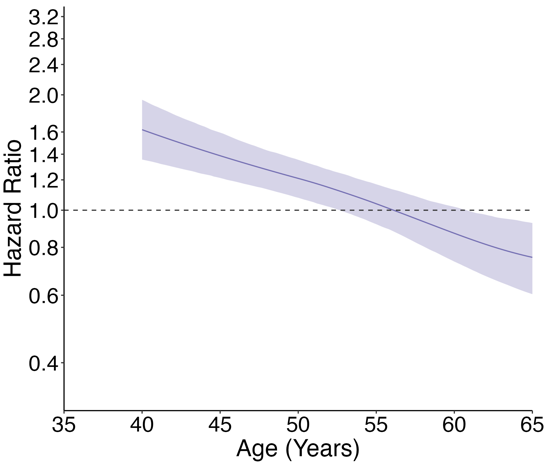 | 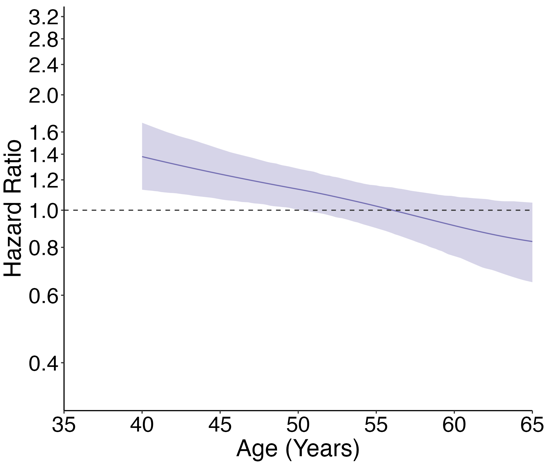 | 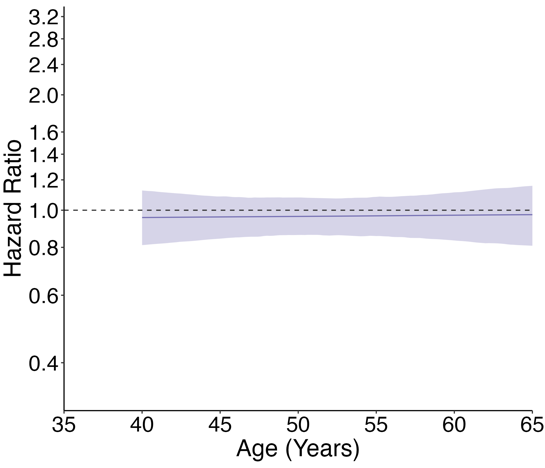 |
| 5. Applying inverse probability of censoring weights | 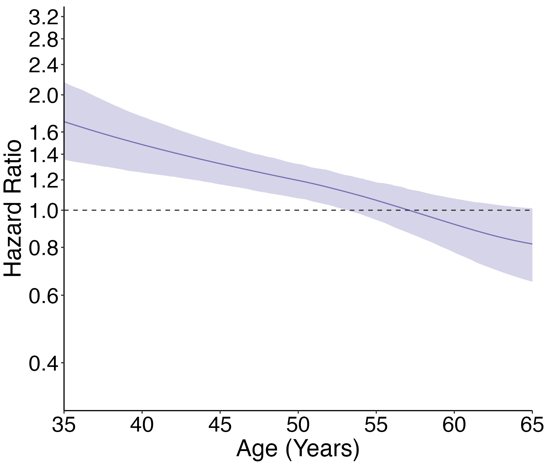 | 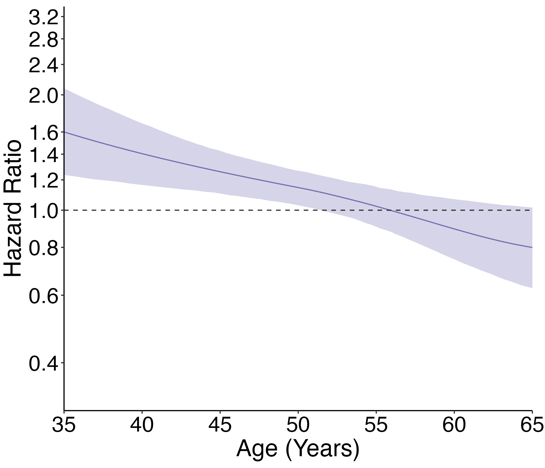 | 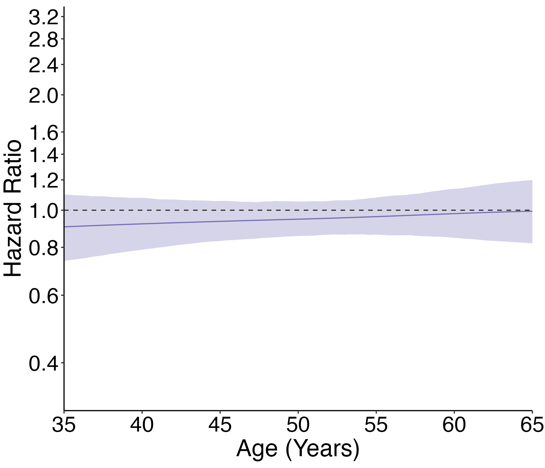 |

Models controlled for birth year, education, smoking, and duration of hormonal contraceptive use.

**Figure S3: Sensitivity analyses of adjusted association between parity and timing of surgical menopause**

|  | A. 0 Births vs. 2 Births | B. 1 Birth vs. 2 Births | C. ≥3 Births vs. 2 Births |
| --- | --- | --- | --- |
| 1. Restricted to women without a history of infertility or recurrent pregnancy losses | 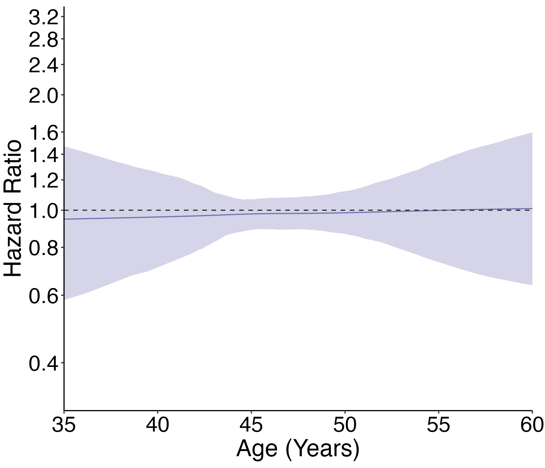 | 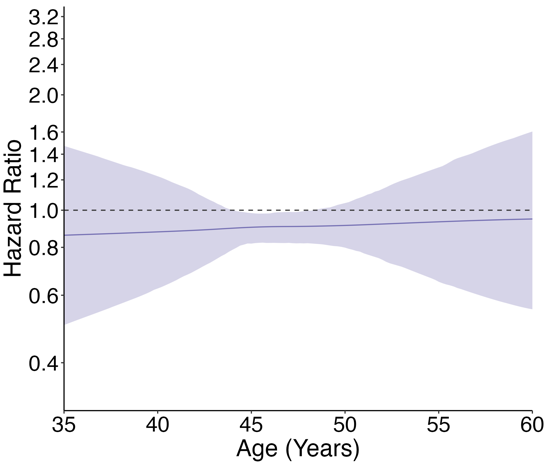 | 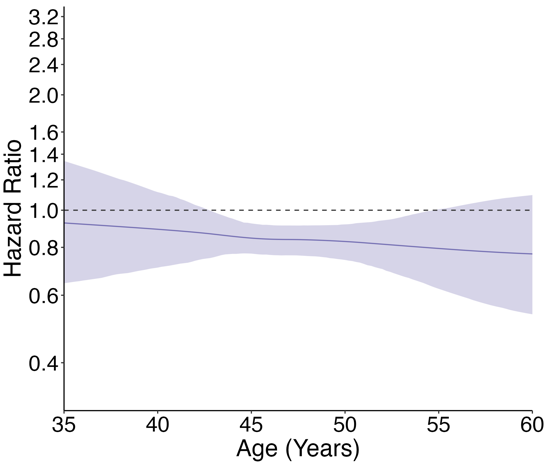 |
| 2. Censored at initiation of premenopausal hormone therapy | 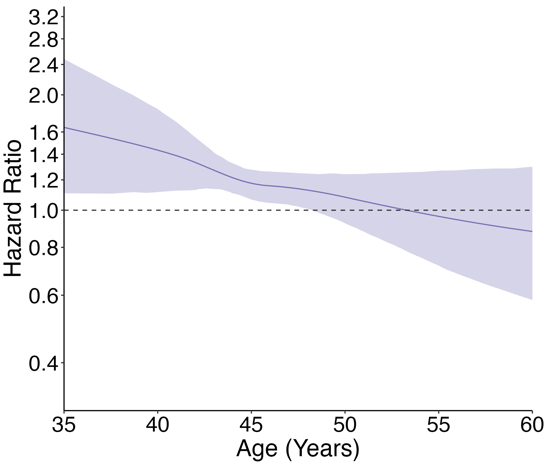 | 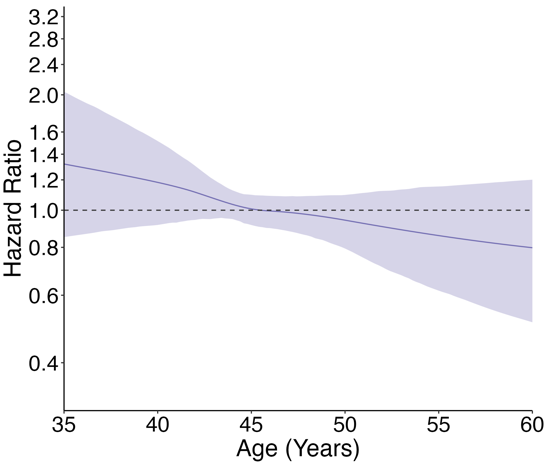 | 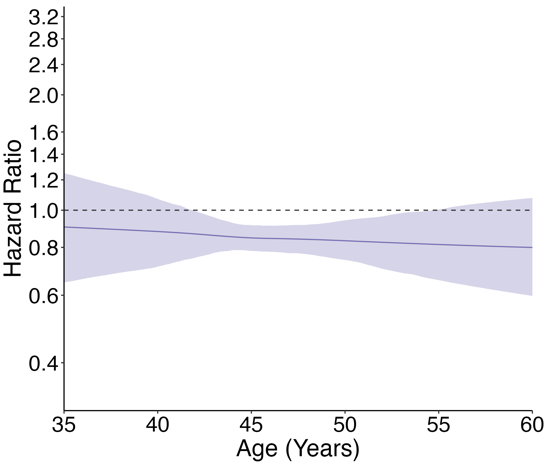 |
| 3. Additionally adjusted for body mass index and chronic medical conditions (diabetes, cardiovascular disease, autoimmune disease) | 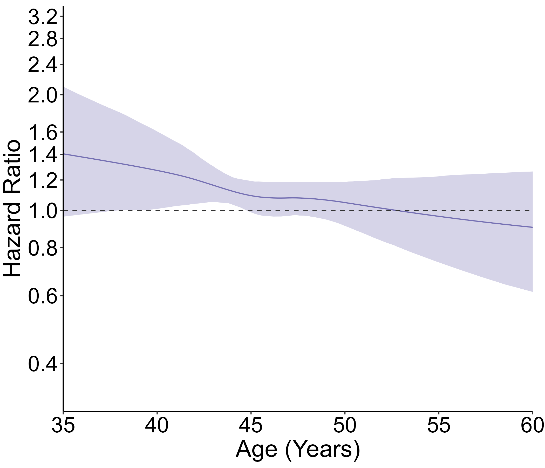 | 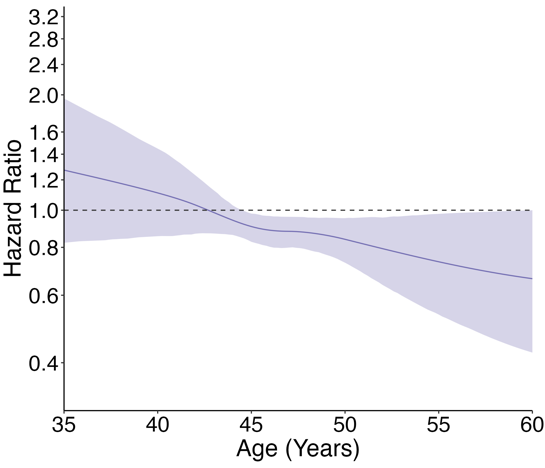 | 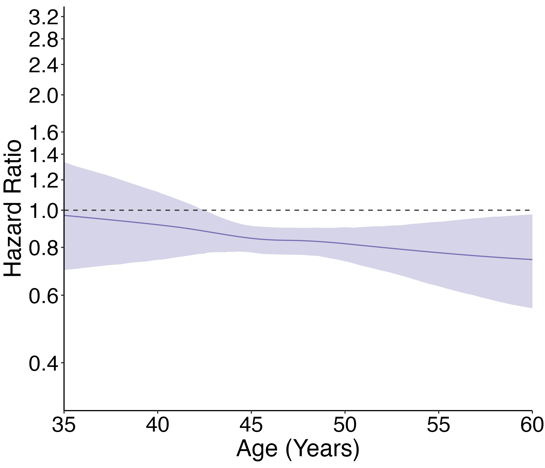 |
| 4. Restricted to menopause >40 years | 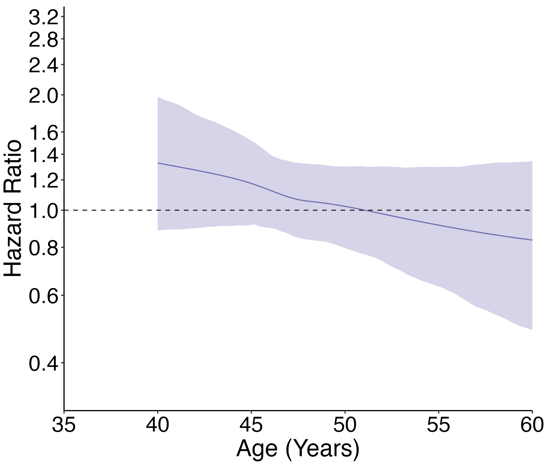 | 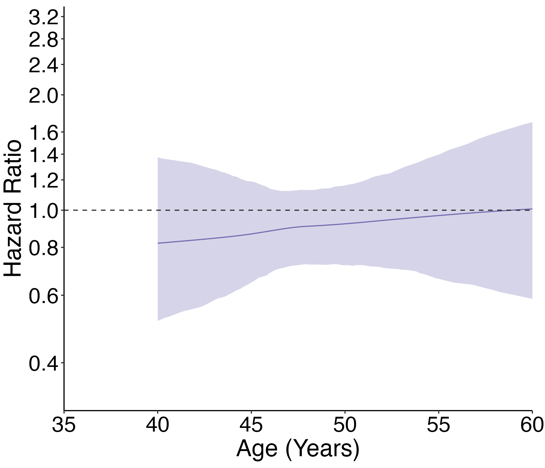 | 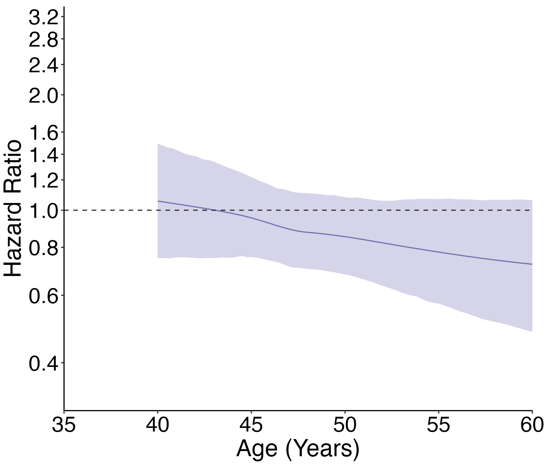 |
| 5. Applying inverse probability of censoring weights | 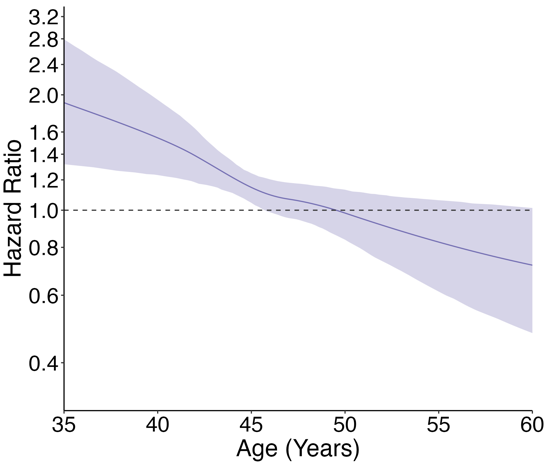 | 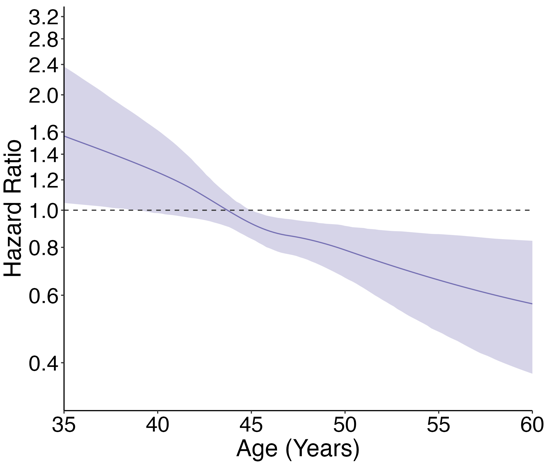 | 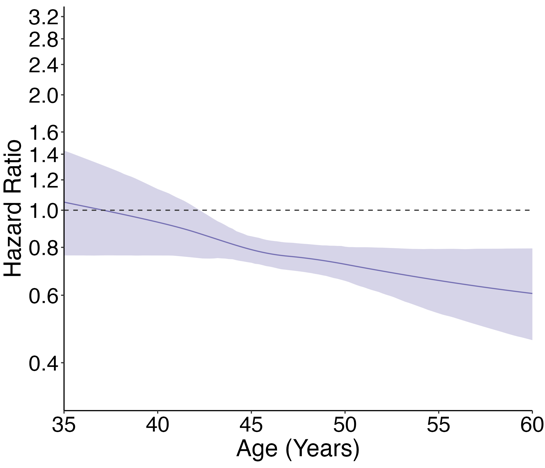 |

Models controlled for birth year, education, smoking, and duration of hormonal contraceptive use.

**Figure S4: Sensitivity analyses of adjusted association between parity and timing of premenopausal hysterectomy**

|  | A. 0 Births vs. 2 Births | B. 1 Birth vs. 2 Births | C. ≥3 Births vs. 2 Births |
| --- | --- | --- | --- |
| 1. Restricted to women without a history of infertility or recurrent pregnancy losses | 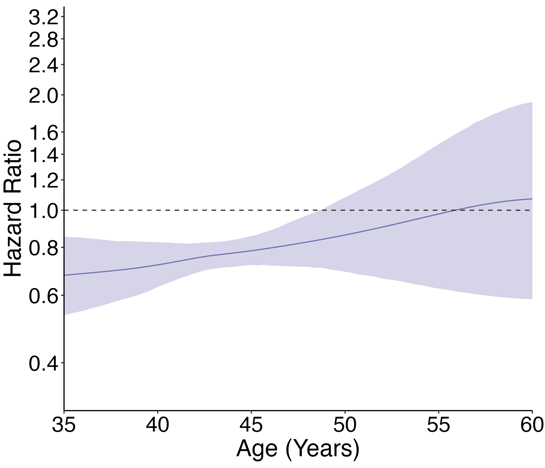 | 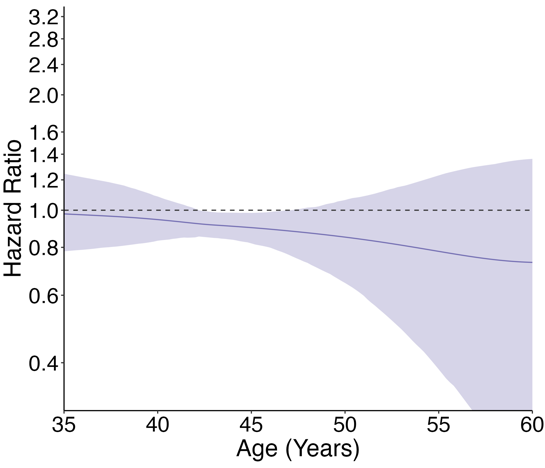 | 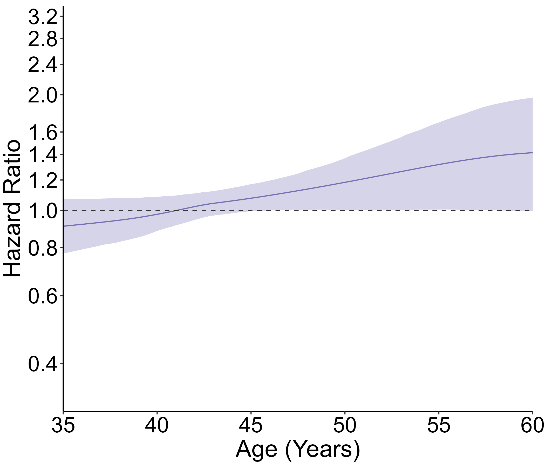 |
| 2. Censored at initiation of premenopausal hormone therapy | 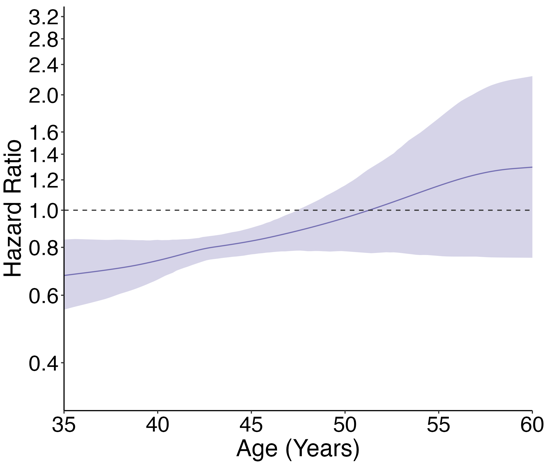 | 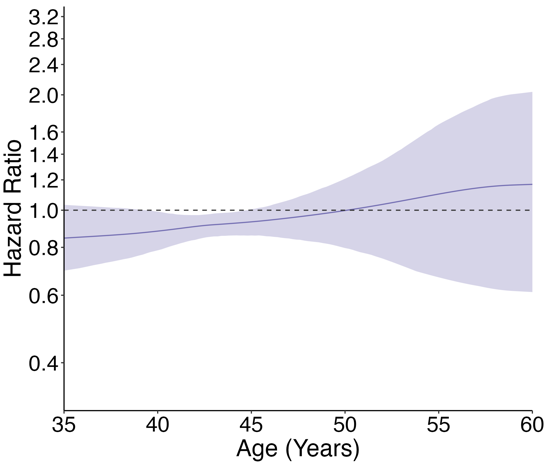 | 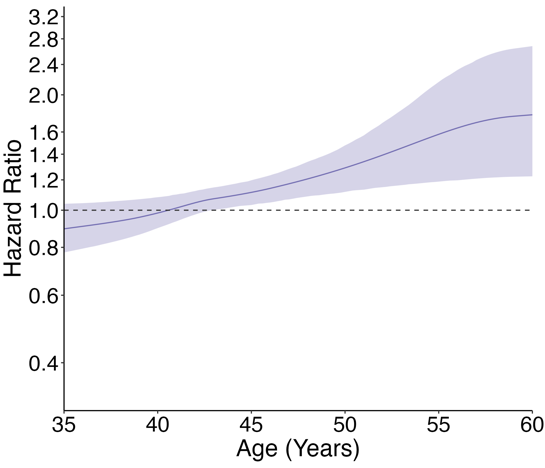 |
| 3. Additionally adjusted for body mass index and chronic medical conditions (diabetes, cardiovascular disease, autoimmune disease) | 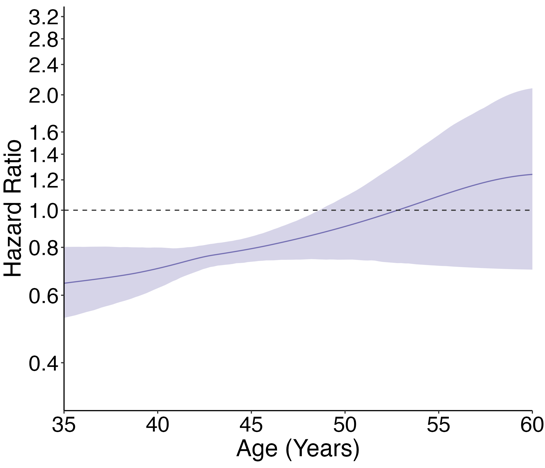 | 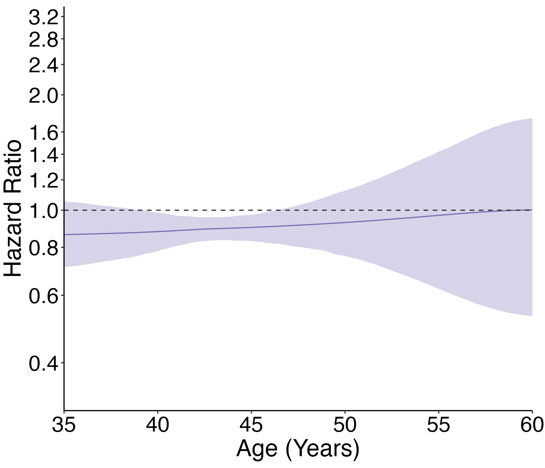 | 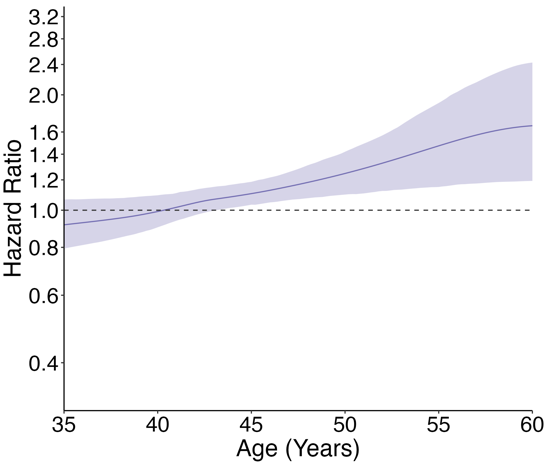 |
| 4. Restricted to menopause >40 years | 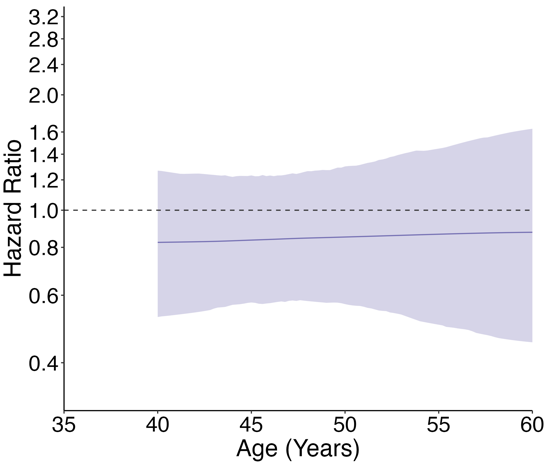 | 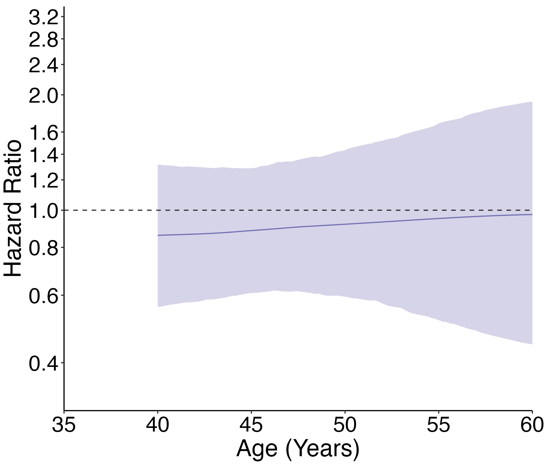 | 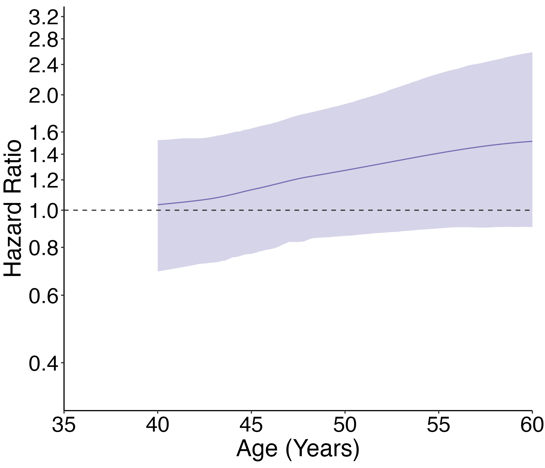 |
| 5. Applying inverse probability of censoring weights | 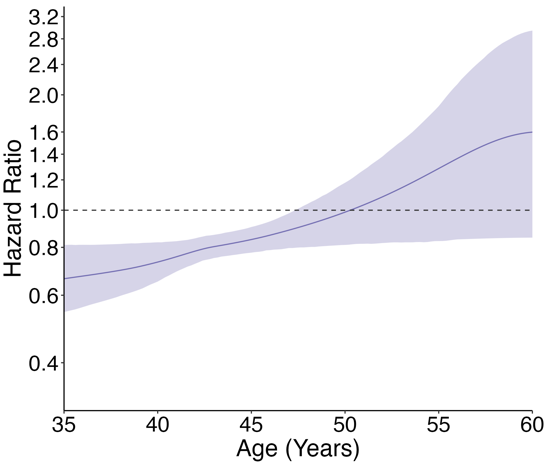 | 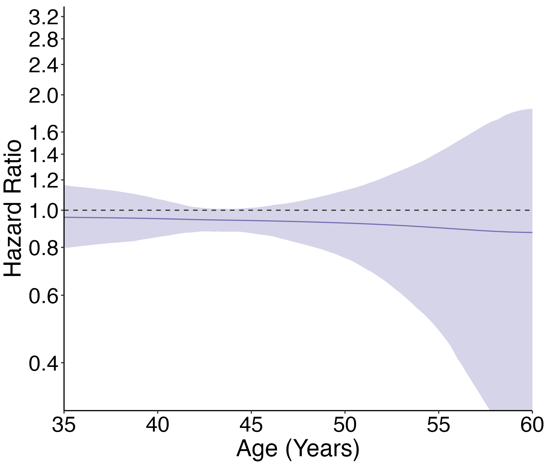 | 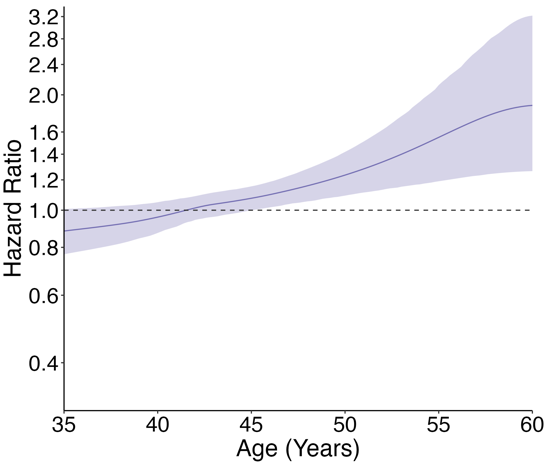 |

Models controlled for birth year, education, smoking, and duration of hormonal contraceptive use.
